# Gene-Expression Signatures Can Distinguish Gastric Cancer Grades and Stages

**DOI:** 10.1371/journal.pone.0017819

**Published:** 2011-03-18

**Authors:** Juan Cui, Fan Li, Guoqing Wang, Xuedong Fang, J. David Puett, Ying Xu

**Affiliations:** 1 Computational Systems Biology Laboratory, Department of Biochemistry and Molecular Biology, and Institute of Bioinformatics, University of Georgia, Athens, Georgia, United States of America; 2 Department of Pathogenobiology, Jilin University, Changchun, Jilin, China; 3 The Second Affiliated Hospital, Jilin University, Changchun, Jilin, China; 4 College of Computer Science and Technology, Jilin University, Changchun, Jilin, China; Ohio State University Medical Center, United States of America

## Abstract

Microarray gene-expression data of 54 paired gastric cancer and adjacent noncancerous gastric tissues were analyzed, with the aim to establish gene signatures for cancer grades (well-, moderately-, poorly- or un-differentiated) and stages (I, II, III and IV), which have been determined by pathologists. Our statistical analysis led to the identification of a number of gene combinations whose expression patterns serve well as signatures of different grades and different stages of gastric cancer. A 19-gene signature was found to have discerning power between high- and low-grade gastric cancers in general, with overall classification accuracy at 79.6%. An expanded 198-gene panel allows the stratification of cancers into four grades and control, giving rise to an overall classification agreement of 74.2% between each grade designated by the pathologists and our prediction. Two signatures for cancer staging, consisting of 10 genes and 9 genes, respectively, provide high classification accuracies at 90.0% and 84.0%, among early-, advanced-stage cancer and control. Functional and pathway analyses on these signature genes reveal the significant relevance of the derived signatures to cancer grades and progression. To the best of our knowledge, this represents the first study on identification of genes whose expression patterns can serve as markers for cancer grades and stages.

## Introduction

Cancer grading is a measure of a cancer's malignancy and aggressiveness. A popular grading system uses four levels of malignancy (G1-G4), reflecting the combined level of cell-appearance abnormality, deviation in growth rate from the normal cells and the degree of invasiveness and dissemination. These pathological measures have been found to be in general concordance with the level of cellular differentiation (American Joint Commission on Cancer) [1]. Hence {G1, G2, G3, G4} are also referred to as well-, moderately-, poorly- and un-differentiated, respectively. As of now, there has not been a universal grading system for all cancers. Instead, different grading systems have been proposed for different cancers. For example, the Gleason system [2] is probably the most well-known for grading adenocarcinoma cells in prostate cancer while the Bloom-Richardson system [3] is used for breast cancer, and the Fuhrman system [4] is used for kidney cancer.

Gastric cancer, the second leading cause for cancer-related death worldwide, is particularly prevalent in Asian countries, including China, Korea and Japan [5]. In the U.S., this asymptomatic disease had ∼21,500 new cases in 2008 along with 10,800 deaths [6]. Unlike other cancers, gastric cancer does not yet have a generally accepted grading scheme. Grading has been mostly done based on rather general cancer-grading guidelines from organizations like the American Joint Commission on Cancer. There are a few systems for classifying gastric cancers into histological subtypes, including those by the Lauren [7], the World Health Organization (WHO) [8] and Goseki, et al. [9], [10], which define subtypes according to the structural features of the cancer, the histopathological appearances of the cells, and the level of mucus, respectively. However, it is largely controversial regarding whether any of these systems is really relevant to the degree of malignance and survivability, thus having not been widely used for grading gastric cancer [11]. The lacking of a well-established grading system for gastric cancer remains as a major obstacle hindering the progress in this field.

We present a computational study herein, aimed to identify a set of genes whose expression patterns can well distinguish among gastric cancers of different grades, like Oncotype DX, a 21-gene panel for identifying low-risk breast cancer [12]. These genes, whose expression patterns distinguish gastric cancers of different grades, provide useful information towards developing a gene expression-based grading system for gastric cancer. In addition, we also present our findings on the gene expression patterns common to cancers at different developmental stages, potentially serving as molecular signatures for gastric cancer staging.

## Results

### A. Identification of genes with expression changes correlated with cancer grades

**Figure 1:**
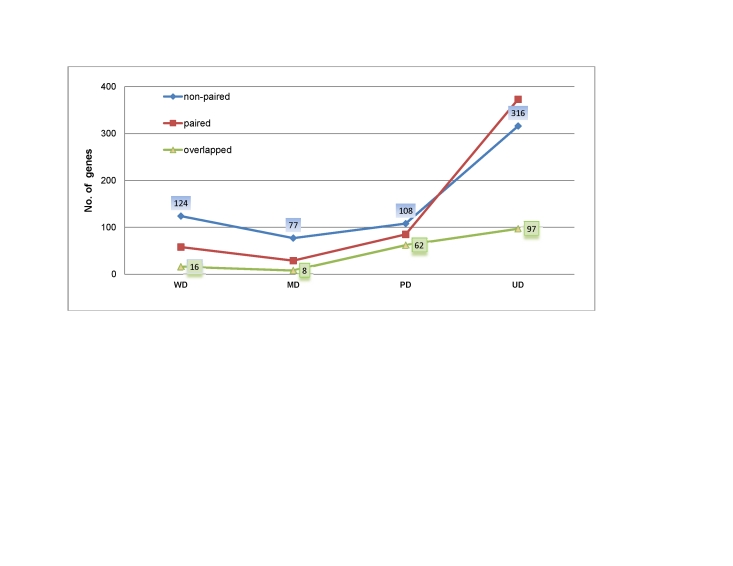
Relationship between cancer grades and the number of differentially expressed genes, with fold-change (FC) > = 2 and *P*-value <0.05 by Wilcoxon signed–rank test (blue), using paired sample information, and fold-change test defined in this study (red), without using paired-sample information. The green plot shows the overlapped identification between these two strategies.

17,800 human genes were profiled in this study, using Affymatrix Exon Arrays. Out of the 54 cancer samples, 8 are well differentiated (WD), 9 moderately differentiated (MD), 35 poorly differentiated (PD) and 2 undifferentiated (UD). A total of 452 genes were found to be differentially expressed as determined using the following criteria: the expression levels in cancer and the corresponding control tissue show at least 2-fold change, and the statistical significance, *P*-value, of having this level of expression change is <0.05 (see Material and Methods; gene names are listed in Table S1). Among the 452 genes, 97 uniquely in UD, 62 in PD, 8 in MD and 16 uniquely in WD represent a *core set* of differentially expressed genes, which are consistently identified by applying different classification strategies using the paired-sample information or not. This set includes genes exhibiting the most consistent expression change (over 2-fold) in cancer *versus* control tissues, which were deemed to be differentially expressed genes with high reliability, derived through multiple statistical tests. In contrast, the whole set of 452 genes represent an extended set. We noted that there is a general trend that the number of the differentially expressed genes increases as a gastric cancer, relative to normal tissue, is more poorly differentiated, as shown in Figure 1. This observation is in agreement with our general knowledge that less-differentiated cancers tend to have more differentially expressed genes and are more aggressive; the exception for WD, as shown in Figure 1, might reflect the small sizes of the WD and the MD groups.

**Figure 2:**
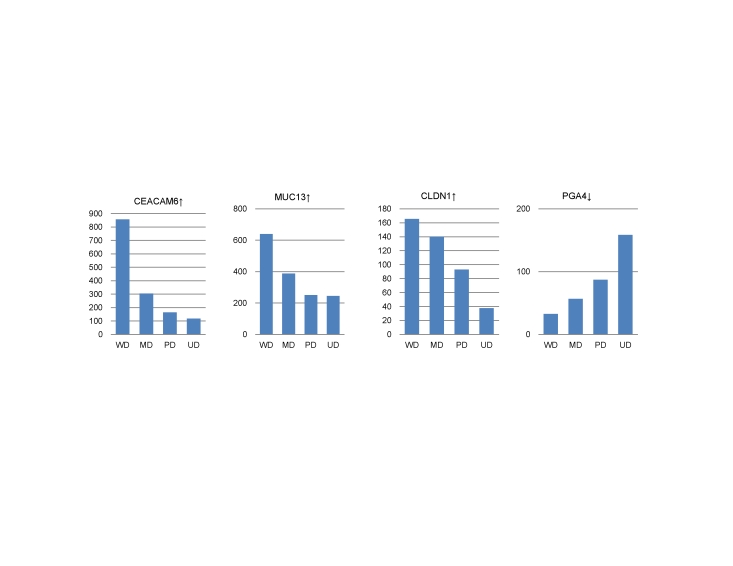
Correlation between gene expression levels and cancer grades of four genes. (“↑” and “↓” denote up- and down-regulation in cancer *versus* reference tissues, respectively.)

We then checked if some genes may have their expression changes correlate with the cancer grades. To do this, we have calculated the Spearman correlation coefficient (CC) between the average expression of each gene across all samples of each grade and the four cancer grades. It was found that the expression changes of 99 genes correlate perfectly with the grades WD-MD-PD-UD (|*CC*| = 1, *P*-value<0.05) (see details in Table S2). Among these genes are *POF1B*, *MET*, *CEACAM6*, *ZNF367*, *GKN1*, *LIPF*, *SLC5A5*, *MUC13*, *CLDN1*, *MMP7 and ATP4A*, which are all known to be cancer related. Figure 2 shows four examples with either positive or negative correlations. Among them, *MUC13* has been reported as a good marker for the level of differentiation of gastrointestinal mucosa [13]. Increased MUC13 expression has been found to induce morphological changes, including scattering of cells through interference with the function of cell adhesion molecules [14]; thus, an increased expression along with differentiation may indicate enhanced cell-cell adhesion.

We noted that genes with their expression changes correlated with cancer grades are highly enriched among secreted or membrane proteins (P-value <0.05), which participate in multiple signaling pathways such as ErbB, FAS, NOD-like receptor, PPAR and Wnt signaling, as well as cell adhesion molecules (CAMs) and tight junctions. This is not surprising since these pathways are essentially involved in cell growth and cell death, as well as cancer metastasis. Such changes in gene-expression patterns of these pathways, involved in signal transduction and extracellular communication, may provide clues about cancer progression.

### B. Identification of gene signatures for cancer grades

We have examined the 452 differentially-expressed genes, aiming to identify genes whose expression patterns can, with good accuracy and reliability, distinguish gastric cancers of different grades. The classification analysis (see Methods) was first conducted between two cancer groups (highly and poorly differentiated), and then extended to five groups, namely four cancer grades and the control. A support vector machine (SVM)-based regressive feature elimination approach was applied, using a linear kernel for cancer classification (see Methods).

At the end, a 19-gene group was identified which can distinguish between highly and poorly differentiated cancers with an overall agreement at 79.2%, based on the expression fold-change in cancer *versus* control tissues. Similarly, a 198-gene group can distinguish among the four different cancer grades and the control group according to their gene expression, giving rise to 74.2% overall classification accuracy. Both gene sets were chosen based on a majority voting (at least 70% consistency) scheme from the classification results on 500 sets randomly sampled from the 54 sample sets, along with their significance ranking (see Methods for details).

The 19-gene signature consists of ADIPOQ, COL6A3, TNS1, SCN7A, DES, VIL1, COL3A1, C2orf40, SMYD1, ACTG2, MEIS1, C7, GPR174, SHCBP1, DUSP1, DNAJB5, HIATL1, IL17RB, and FAT. A close look at the functional annotation of these genes revealed that their protein products are involved in cell growth and differentiation (IL17RB, SMYD1, SHCBP1), cell motility (ACTG2), angiogenesis and tissue remodeling (ADIPOQ), carcinogenesis (ECRG4), matrix protein synthesis (COL3A1, COL6A3), and others like G protein-coupled receptor 174 (GPR174), brush border cytoskeleton (VIL1), membrane attack complex (C7), and sodium channel (SCn7A).

17 out of the 19 genes, plus an additional 181 genes, form a 198-gene group whose expression pattern can distinguish the four cancer grades and the control. Their functions cover cell division, immune response, signal transduction and transcription regulation, in addition to the aforementioned categories. Overall, 39 out of 99 grade-correlated genes are part of this 198-gene signature, including CLDN1, MUC13, VIL1, HIATL1, CDCA7, HIST1H2BM and FAT (see the full list in Table S3).

**Figure 3:**
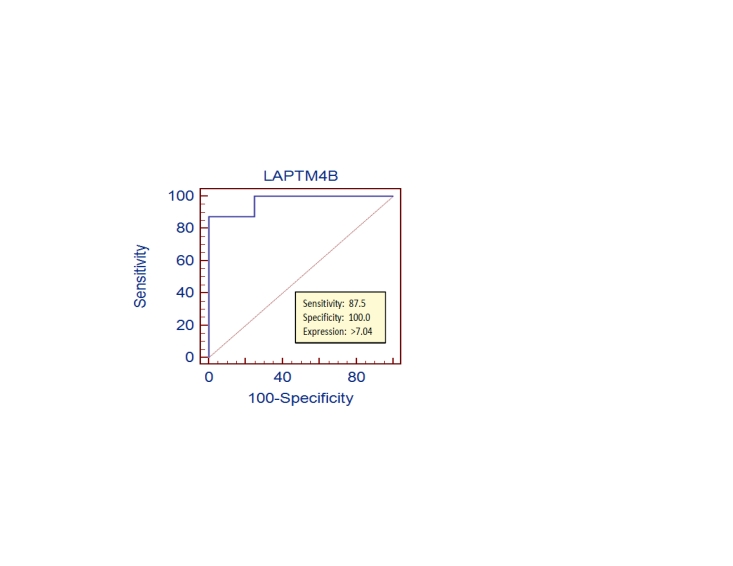
The ROC curve of LAPTM4B as a discriminator between cancer and control samples in the WD group (with AUC of 0.97).

In addition to this catch-all signature for five-way classification, we also identified and analyzed grade-specific gene signatures for each cancer grade. For example, LAPTM4B is one such representative. This gene gives high classification accuracy for caner and control samples in the WD group with the AUC (area under curve)  = 0.97 (Figure 3). Using 7.04 as the expression cutoff, this gene can well distinguish cancer from the control samples in the WD group with sensitivity  = 87.5% and specificity  = 100%. This result is not surprising since it is known that LAPTM4B is essential for cell growth and survival, and its up-regulation has been found to be correlated with the level of differentiation of hepatocellular carcinoma [15]. In total, 40 such signature genes are found specifically for the WD group; 18, 20 and 255 genes are specific to the MD, PD and UD group, respectively (see details in Table S4).

We have also identified single gene discriminators for each grade group against the rest of the samples, including the control, as summarized in Table 1. For instance, the signatures for the PD group include the up-regulated genes, *MYO1B* for WD; *GKN2* for MD; *CTSA* for PD; and a down-regulated gene, *RHOJ,* for the UD group. These single-gene discriminators show significant AUCs, ranging from 0.76 to 0.99, while the overall classification accuracies obtained by 5-fold cross-validation range from 70.0% to 97.0% for different groups. A subsequent search for *k*-gene combinations (k = 2, 3, 4) for each cancer group by exhaustively going through all the combinations of *k*-gene groups also identified.

**Table 1:** The top three discriminative genes for each grade (against the rest), through classification analysis based on both their expression levels (*P-value is obtained by Wilcoxon signed–rank test; “↓” denotes a down-regulated gene; REL means “raw expression level”).

Subtype	REL-based signatures			
	Genes	AUC	*P-value	ClassificationAcc. (sen./spe.)%
**WD**	MYO1B	0.85	1.07E-03	81.5(75.0/82.6)
	MET	0.84	1.33E-03	80.9 (62.5/84.1)
	EDARADD↓	0.83	1.70E-03	72.3(85.7/69.7)
**MD**	GKN2	0.77	1.42E-02	74.3(77.8/74.0)
	SPP1	0.83	4.94E-04	75.7(66.7/76.3)
	PDIA2	0.87	7.82E-04	70.2(77.8/68.7)
**PD**	CTSA	0.76	1.21E-06	75.8 (87.5/62.0)
	ADAMTS12	0.79	6.81E-08	75.0 (75.0/75.0)
	CST2	0.78	1.77E-07	74.2 (78.1/69.4)
**UD**	COTL1	0.99	3.33E-01	96.4(100/96.4)
	RHOJ↓	0.99	3.33E-01	97.1(100/97.1)
	TNFRSF1B	0.99	3.33E-01	97.1(100/97.1)

### C. Identification of gene signatures for pathological stage

Using similar analyses to those of the above, we have identified gene signatures for early stage (stage I+II) and advanced stage cancer (stage III+IV). Table 2 highlights the most discriminative single gene markers, with the classification accuracy ranging from 75.0% to 81.4%. Multi-gene signatures were also checked for cancer staging. For example, two signatures were found to be particularly effective in cancer staging, namely a 10-gene group (CPS1+ DEFA5+ DES+ DMN+ GFRA3+ MUC17+ OR9G1+ REEP3+ TMED6+ TTN) and a 9-gene group (DPT+ EIF1AX+ FAM26D+ IFITM2+ LOC401498+ OR2AE1+ PRRG1+ REEP3+ RTKN2), which can distinguish the early and the advanced gastric cancers from the remainder of the samples (including control samples) with agreements of 90.0% and 84.0%, respectively. The overall classification accuracy on the three groups, early, advanced and control, is 71.4%.

**Table 2:** The most discriminative genes identified for staging through classification analysis based on both their expression level and expression fold-change (*P-value is obtained by Wilcoxon signed–rank test; “↓” denotes a down-regulated gene. “–“ is included since the ECMs based on fold-change is applicable to both early and advanced stages; REL means “raw expression level” while EFC means “expression fold-change”).

Stage	REL-based signatures				EFC-based signature			
	REMs	AUC	P-value	ClassificationAcc.(sen./spe.)%	ECMs	AUC	P-value	ClassificationAcc.(sen./spe.)%
**I+II**	CHRM3↓	0.83	3.36E-04	79.3(90.9/67.4)	GNG5	0.86	2.06E-04	83.2(100/64.4)
	PCDH7↓	0.82	3.78E-04	78.9(91.9/66.7)	DKK2	0.74	1.08E-02	78.4(81.8/74.6)
	TACR2	0.78	2.40E-03	78.5(100/56.6)	KIF2B	0.79	2.69E-03	76.8(81.8/71.2)
	SATB2	0.82	4.64E-04	77.0(81.8/72.1)	C3orf20	0.77	4.99E-03	76.8(72.7/81.4)
	LANCL3↓	0.78	2.28E-03	0.77(0.91/0.62)				
	PPA1	0.80	1.14E-03	0.75(0.82/0.69)				
**III+IV**	RTKN2	0.54	2.88E-11	81.4(71.1/88.9)	–		–	–
	PKM2	0.63	2.25E-10	79.3(69.5/86.4)	–		–	–
	B4GALNT2	0.52	5.14E-09	77.8(83.1/74.1)	–		–	–
	MFAP2	0.62	5.73E-09	77.1(66.1/85.2)	–		–	–

A functional analysis on these signature genes revealed something interesting. For example, among the protein products of early-stage signature genes, *GFRA3*, *MUC17*, *OR9G1*, *REEP3 and TMED6* are membrane proteins, mostly receptors that transduce extracellular signals. *DEFA5* is a microbicidal peptide believed to be involved in host defense that is highly expressed in the ileum [16]. *CPS1*, *DES and TTN* are involved in multiple metabolic processes, muscle function and the M phase of the mitotic cell cycle, respectively. We speculate that these signaling- and immune- related genes may represent the early abnormality of tissue cells during oncogenesis in general.

**Figure 4:**
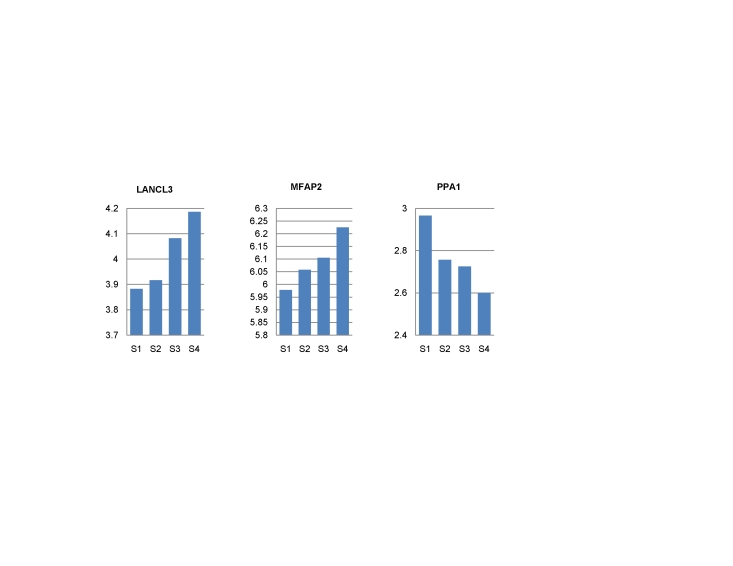
Correlation between gene expression (log transformed) and the pathological stages. (S1–S4 represents four stages from early stage I to advanced stage IV.

A few genes were found to be in both the cancer grading and staging signatures, such as CPS1, DES, GFRA3, TMED6 and DPT, indicating some biological relevance between cancer differentiation and progression. We then examined whether the gene expression of staging signatures are associated with pathological stages. Among them, those highly correlated with different pathological stages are *LANCL3*, *MFAP2 and PPA1* (Figure 4), showing consistent up- and down-regulation, respectively, along with cancer progression.

### D. Identification of differentially-expressed genes independent of cancer grades and stages

In addition to the differential expression specific to certain subgroups of gastric cancer, we also examined if some genes are differentially expressed in gastric cancer in general, regardless of grades and stages. 62 such genes were found with consistent differential expression by at least 2-fold changes in cancer *versus* corresponding reference tissues. We noted that they are mostly involved in extracellular processes such as focal adhesion, CAMs, tight junction, cytokine-cytokine receptor interaction and ECM-receptor interaction, the plasminogen activation cascade, as well as signaling pathways including Wnt signaling and Integrin signaling, which are closely relevant to cell growth and cell proliferation control. Searching against our in-house database (http://bioinfosrv1.bmb.uga.edu/DMarker/) which includes public microarray datasets from GEO [17], Oncomine [18] and SMD [19], covering over 53 human diseases including cancer, we found that the differential expression patterns of 15 genes are highly specific to gastric cancer, such as GKN2, CLDN7, THY1, GIF and PGA4, while most others are general to multiple cancer types. For example, the most general ones include a few members of the collagen gene family (COL1A2, COL3A1 and COL1A1), the carcinoembryonic antigen–related cell adhesion molecule (CEACAM6), matrix metalloproteinases (MMP1, MMP7 and MMP12), topoisomerase (TOP2A) and secreted phosphoprotein (SPP1).

**Figure 5:**
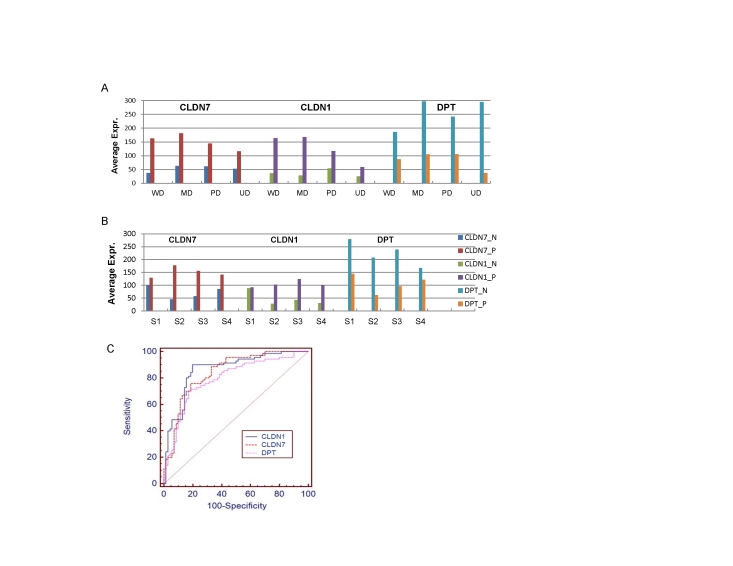
Average expression of three genes (CLDN7, CLDN1 and DPT) in cancer and normal samples, respectively. (A) for each subtype (WD, MD, PD, UD); (B) for each stage (stage I, II, III and IV); and (C) the ROC curve shows the discerning power of each gene for classification of cancer *versus* normal samples (AUCs of CLDN1, CLDN6 and DPT are 0.86, 0.84 and 0.79, respectively, with a significance level of P = 0.0001).

Only three, *CLDN7*, *CLDN1 and DPT*, of these genes are significantly differentiated in all grades or stages of gastric cancer. We can see from Figure 5A and 5B that both *CLDN7 and CLDN1* are highly expressed in cancer *versus* control samples across all grades and stages, with a moderate increase in early cancer tissues, while *DPT* was down-regulated across all these groups. The consistent expression pattern across all the cancer subgroups may indicate that these genes participate in many major biological pathways involved in cancer formation and progression. As is well known, the two claudin proteins, claudin-1 and claudin-7, are integral membrane proteins crucial to formation of tight junctions, maintaining cell-to-cell adhesion and regulating paracellular and transcellular transport of solutes across human epithelia and endothelia, which are differentially expressed in various cancers such as cervical neoplasia [20], renal carcinoma [21] and an intestinal type of gastric cancer [22]. Dermatopontin (*DPT*) is an extracellular matrix protein serving as a communication link between the dermal fibroblast cell surface and its extracellular matrix. Its reduced expression has also been found in both uterine leiomyomas and keloids [23]. The ROC shown in Figure 5C indicates that these genes could possibly be used as effective markers for gastric cancer diagnosis in general.

### E. Verification of the identified signatures on public datasets

**Figure 6:**
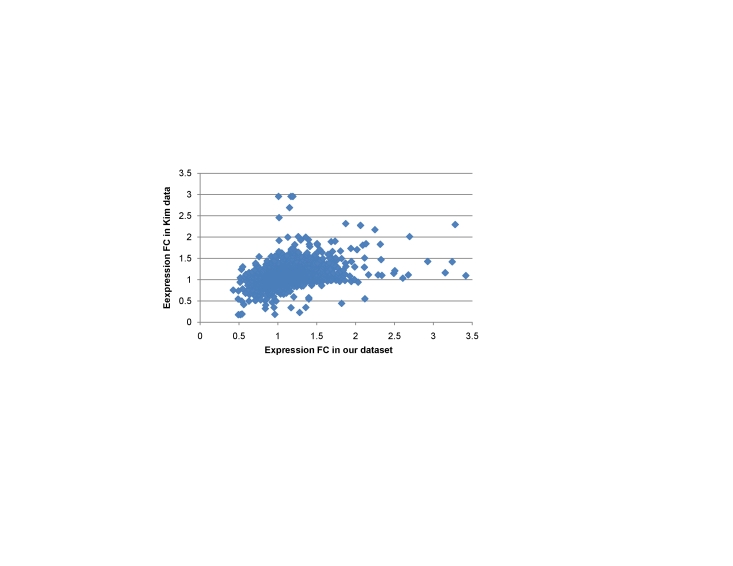
Distribution of expression differentials between out data and *Kim* dataset.

The expression patterns of our identified signature genes were checked against two public datasets, namely, the *Kim* and *Takeno* datasets (see Materials and Methods), to determine the generality of these gene signatures. As shown in Figure 6, the distribution of expression differentials between our data and the *Kim* dataset is significantly concordant, indicating that the general applicability of our identified markers. Out of 19 and 12 overlapped genes from the above-identified grades-correlated and stage-correlated gene list, 10 and 5 show similar expression patterns across cancers of G1-2/G3-4 grades and I-IV stages in the *Kim* data, respectively, reflecting a high consistence in expression patterns of these genes among different sample sets.

Overall, our 19-gene signature for cancer grades performed well on the *Kim* data and obtained 78.0% classification accuracy on 5-fold cross validation in terms of distinguishing poorly from highly differentiated cancers. Similarly, the two-stage signatures (10-gene and 9-gene groups) obtained respective accuracies of 84.0% and 76.0% on the *Kim* dataset. The 198-gene signature was not checked since the *Kim* dataset provides only fold-change instead of raw expression data.

Interestingly, we noted that there is moderate correlation between the gene expression of our identified signature groups and cancer recurrence based on the peritoneal relapse information of Takeno's data [24]. Specifically, the four signatures, 19-, 198-, 10- and 9-gene groups, can predict the peritoneal relapse with an overall accuracy of 66.0%, 87.2%, 73.0% and 55.3%, respectively, by distinguishing between the relapse-free and peritoneal-relapse patients in Takeno's study [24].

## Discussion

Microarray gene-expression analyses on gastric cancer have previously identified gene expression patterns for prognosis prediction [25], [26] and general cancer diagnosis [27], [28] (as reviewed in Table S6) but none for gastric cancer subtyping or grading. Here, we presented an analysis on 54 pairs of cancer and adjacent reference tissues from the same number of gastric cancer patients, and identified molecular signatures for cancer grades and stages.

It is known that different classification and gene selection analyses may lead to different gene signatures, posing a serious issue about the stability and usefulness of the selected gene signatures. To deal with this issue, we have applied exhaustive searches for k-gene signatures (k< = 4) coupled with a robust feature selection procedure with majority voting for k>4, which ensures the stability of the identified signature genes. On the other hand, due to the complex nature of cancer gene-expression data, a general belief has been that different classification techniques may give rise to different signatures but of equal importance as they may correspond to different pathways associated with different aspects of a cancer. In addition to these technical variances, the limited sample size and the heterogeneity existing among the cancer subgroups are noted as other major factors affecting the selected markers.

In conclusion, we have demonstrated herein that gene expression patterns can be used as effective signatures for gastric cancer grading and staging, as well as prognostic prediction. Two types of signatures were proposed to serve different diagnostic purposes, each showing a certain relevance to cancer malignance and cancer progression. Such attempts of using molecular grade-and stage-signatures are expected to significantly benefit the development of personalized medicine and may lead to new serum markers.

## Materials and Methods

### Tissue Samples

Samples were taken from primary malignant gastric cancers from non-treated patients during the initial surgical procedure at three affiliated hospitals of the Jilin University College of Medicine and Jilin Provincial Cancer Hospital, Changchun, China. For each cancer tissue sample, a matching reference tissue sample was collected from the adjacent noncancerous region that the surgeon resected in order to ensure positive margins. All samples were snap-frozen in liquid nitrogen within 10 minutes after excision and stored at -196C until RNA extraction. For RNA isolation, 100 µm sections of each sample were used.

All medical records and cancer sections were examined by a surgical pathologist, and the histological diagnosis and TNM classification were made according to Worldwide Health Organization (WHO) criteria and the classification system of the International Union against Cancer. The reference samples were subjected to a meticulous histologic analysis to guarantee the complete absence of cancer cells. Written informed consent was obtained from all patients, which was approved by the Institutional Review Board at the University of Georgia, Athens, Georgia, USA and by the Chinese IRB overseeing human subjects at Jilin University College of Medicine and the Jilin Provincial Cancer Hospital, Changchun, China.

Detailed patient information such as age, gender, histological type, differential grade, pathologic stage and history of using alcohol/smoking is listed in Table S5.

### Microarray experiments

The RNA samples were analyzed using the GeneChip Human Exon 1.0 ST (Affymetrix), following the protocol detailed in the Genechip Expression Analysis Technical Manual (P/N 900223) for the array experiment and an earlier report [29]. The microarrays were scanned using the GeneChip® Scanner 3000 with GeneChip® Operating Software (GCOS). All data is MIAME compliant and the raw data has been deposited in GEO database (ID: GSE27342).

### Microarray Data Analysis

Gene expression results were summarized based on raw probe intensities using the Robust Multichip Average [30] and the APT package (http://www.affymetrix.com/partnerSupplementaryprograms/programs/developer/tools/powertools.affx), following three main steps including background correction, quantile normalization and log2-transformation. Genes having very low expression in both cancer and reference samples were removed; specifically, a gene was removed if its *maximum(Expr.cancer, Expr.normal)* was below 4 (normalized signal intensity).

Two different strategies were applied for assessing gene significance, depending upon what conditions were compared and whether paired or unpaired samples should be used. For comparison of cancers against control sample groups, unpaired tests were conducted to investigate if two groups of expression are different, while paired tests were applied to examine the consistency of expression changes across all pairs. In addition to the Wilcoxon signed–rank test, we also applied another simple statistical test to detect genes with consistent differential expression in cancer *versus* reference tissues, as follows. For each gene, *K_exp_*, the number of pairs of cancer/reference tissues whose expression fold-change (FC) is larger than *k* (e.g. *k* = 2) was examined; if the P-value for the observed *K_exp_* was less than 0.05, the gene was considered to be differentially expressed in the majority of the cancer and reference tissue pairs (see the supporting information). Our calculated P-value was not adjusted on the multiple hypotheses testing in order to avoid any loss of genes that may be potentially effective in the subsequent classification step.

### Gene selection and classification

For k-gene signatures (k< = 4), we conducted an exhaustive search for all the k-gene combinations among the differentially expressed genes, identified from the previous step, using a linear SVM-based classification approach, and the overall accuracy was evaluated using 5-fold cross-validation. For k>4, a different approach using a heuristic search was applied since the exhaustive search is too time-consuming to be practical for our problem. The details are as follows.

The whole expression data set was randomly split into training and test sets, each containing half of the samples. This was repeated for 500 times to generate 500 sets of training/test data for classification. A linear SVM was used for training a classifier [31], [32]. It constructs a hyper-plane that separates two different classes of feature vectors with a maximum margin. This hyper-plane is constructed by finding a vector w and a variable b that minimize 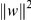, which satisfies the following conditions:


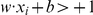
, for
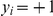
(cancer samples) and 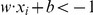, 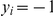
(normal samples). Here, 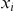
is a feature vector, 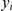
is the group index, w is a vector normal to the hyper-plane, 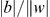
is the distance from the hyper-plane to the origin and 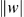 is the Euclidean norm of w. After the determination of w and b values, a given vector x can be classified by using 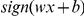; a positive or negative value indicates that the vector x belongs to the positive or negative class, respectively. Gene signatures of each training set were selected by using the recursive feature elimination procedure (RFE), which is a wrapper that selects predictor genes by eliminating non-predictor genes according to a gene-ranking function generated from the classification system [33]. The ranking criterion is based on the change in the objective function upon removing each gene. To improve the efficiency of training, this objective function is represented as a cost function *J* for the *i*-th feature, computed by using the training set only. When a gene is removed or its weight w_i_ is reduced to zero, the change in the cost function *J(i)* is given by 
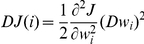
. The case of 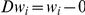 corresponds to the removal of the *i*-th gene. The change in the cost function indicates the contribution of the gene to the decision function and serves as an indicator of gene ranking.

The 500 training/test sets were randomly divided into 10 sample groups. Every sample group was then used to derive a signature, based on majority voting and evaluation of gene-ranking consistency across the 50 training and test sets. The 10 different signatures derived from the 10 groups were compared to assess the level of consistency among the selected genes. In each group, subsets of genes were selected by RFE-SVM from each training set, and the performance on the subsets was evaluated from the associated test set. To derive a gene ranking criterion consistent for all iterations, a RFE ranking function at every iteration step was derived from an SVM classifier that gave the best average classification accuracy over the 50 test sets.

### Public microarray data of gastric cancer

Two public microarray datasets were downloaded from the GEO database for comparative studies, the *Kim* (GSE3438) and the *Takeno* (GSE15081) datasets. The first one [34] includes gene expression of 50 gastric cancer patients (from Korea) at different stages and level of differentiation, which was used to check the consistency of our identified signatures. The Takeno data [24] includes 141 primary gastric cancer tissues after curative surgery, with follow-up peritoneal relapse information. These datasets provide the normalized log2 ratio of tumor and normal expression.

## Supporting Information

Table S1 Statistics of 452 genes that are differentially expressed in any of the four grades group, determined using the following criteria: expression levels in cancer and the corresponding control tissue show at least 2-fold change, and the cutoff for statistical significance of having this level of expression change is *P*-value <0.05.
(XLSX)

Table S2 99 genes have their expression changes perfectly correlate with the grades WD-MD-PD-UD (|*CC*| = 1, *p*-value<0.05).
(XLSX)

Table S3 List of the gene names of the 198-gene signature, among which 39 are grade-correlated gene. CC: Correlation Coefficient.
(XLSX)

Table S4 List of 40 signature genes that are found specifically for the WD group; 18, 20 and 255 genes are specific to the MD, PD and UD group, respectively.
(XLSX)

Table S5 (a) Patient statistics. (b) Detailed information of samples collected in our study (N.B.: information on age, smoking, alcohol consumption, and weight are not complete for all 54 patients, as denoted as “-” in (b); Under smoking and alcohol, “0” and “1” indicate no and yes, respectively).
(DOCX)

Table S6 Recent key findings of biomarkers by transcriptomic and proteomic studies on gastric cancer.
(DOCX)
