# Supplementary material for: Gene-Expression Signatures Can Distinguish Gastric Cancer Grades and Stages
Source: PLoS One. 2011 Mar 18;6(3):e17819. doi: 10.1371/journal.pone.0017819 (PMC3060867; doi:10.1371/journal.pone.0017819)
Supplement: Table S5 — (a) Patient statistics. (b) Detailed information of samples collected in our study (N.B.: information on age, smoking, alcohol consumption, and weight are not complete for all 54 patients, as denoted as “-” in (b); Under smoking and alcohol, “0” and “1” indicate no and yes, respectively). (DOCX) [file pone.0017819.s005.docx]

Supplementary Table 5: (a) Patient statistics. (b) Detailed information of samples collected in our study (N.B.: information on age, smoking, alcohol consumption, and weight are not complete for all 54 patients, as denoted as “-” in (b); Under smoking and alcohol, “0” and “1” indicate no and yes, respectively )

(a)

| **Characters** | | **Patients** | |
| --- | --- | --- | --- |
|  |  | **No. of cases** | **Percentage (%)** |
| Gender | Female | 22 | 40.7 |
|  | Male | 32 | 59.3 |
| Grade | G1 (WD) | 8 | 14.8 |
|  | G2 (MD) | 9 | 16.7 |
|  | G3 (PD) | 35 | 64.8 |
|  | G4 (UD) | 2 | 3.7 |
| Stage | I | 3 | 5.6 |
|  | II | 6 | 11.1 |
|  | III | 41 | 75.9 |
|  | IV | 4 | 7.4 |
| Age | >=55 | 37 | 68.5 |
|  | <55 | 17 | 31.5 |
| Smoking | Yes | 15 | 32.6 |
|  | No | 31 | 67.4 |
| Alcohol | Yes | 9 | 18.8 |
|  | No | 37 | 77.1 |

(b)

| **Patient ID** | **Age** | **Gender** | **Histologic Type** | **Grade** | **Stage** | **Smoking** | **Alcohol** | **Weight** |
| --- | --- | --- | --- | --- | --- | --- | --- | --- |
| 1 | 62 | F | WD | G1 | IIIA | 0 | 0 | 60 |
| 2 | 73 | M | WD | G1 | IB | 0 | 0 | 63 |
| 3 | 47 | M | WD | G1 | IB | 1 | 1 | 65 |
| 4 | 59 | M | WD | G1 | III | 1 | 1 | 51 |
| 5 | 49 | M | WD | G1 | III | 1 | 1 | 60 |
| 6 | 56 | F | WD | G1 | IIIA | 0 | 0 | 45 |
| 7 | 43 | F | WD | G1 | III | 0 | 0 | 55 |
| 8 | 62 | F | WD | G1 | IV | - | - | - |
| 9 | 54 | F | MD | G2 | III | 0 | 0 | 70 |
| 10 | 53 | M | MD | G2 | IIIB | 0 | 0 | 60 |
| 11 | 51 | M | MD | G2 | IIIB | 1 | 0 | - |
| 12 | 41 | M | MD | G2 | II | - | - | - |
| 13 | 68 | M | MD | G2 | IV | 0 | 0 | 48 |
| 14 | 71 | F | MD | G2 | III | 0 | 0 | 42 |
| 15 | 65 | M | MD | G2 | IIIA | 0 | 0 | 70 |
| 16 | 55 | M | MD | G2 | III | 0 | 0 | 69 |
| 17 | 55 | M | MD | G2 | IIIB | 0 | 0 | 74 |
| 18 | 63 | M | PD | G3 | IIIB | 1 | 1 | - |
| 19 | 56 | M | PD | G3 | IIIB | 1 | 1 | - |
| 20 | 71 | M | PD | G3 | IIIB | 1 | 0 | - |
| 21 | 55 | F | PD | G3 | IIIB | 0 | 0 | 63 |
| 22 | 64 | M | PD | G3 | IIIB | 0 | 0 | 55 |
| 23 | 53 | F | PD | G3 | IIIB | 0 | 0 | 77 |
| 24 | 56 | M | PD | G3 | IIIB | 1 | 0 | 55 |
| 25 | 53 | M | PD | G3 | III | 0 | 0 | 62 |
| 26 | 71 | M | PD | G3 | III | 0 | 0 | 60 |
| 27 | 58 | M | PD | G3 | III | 0 | 0 | 50 |
| 28 | 42 | M | PD | G3 | IB | 0 | 0 | 52 |
| 29 | 65 | F | PD | G3 | IIIA | 0 | 0 | - |
| 30 | 50 | M | PD | G3 | III | 1 | 0 | 47 |
| 31 | 59 | M | PD | G3 | III | 0 | 0 | 57 |
| 32 | 75 | M | PD | G3 | III | 0 | 0 | 65 |
| 33 | 40 | M | PD | G3 | III | 0 | 1 | 80 |
| 34 | 51 | F | PD | G3 | III | 1 | 0 | 52 |
| 35 | 36 | M | PD | G3 | IIIA | 1 | 0 | 60 |
| 36 | 67 | F | PD | G3 | IV | 0 | 0 | 48 |
| 37 | 65 | M | PD | G3 | III | 0 | 1 | 50 |
| 38 | 65 | F | PD | G3 | IIIA | 0 | 0 | 53 |
| 39 | 53 | F | PD | G3 | IIIA | 1 | 0 | 60 |
| 40 | 60 | F | PD | G3 | IIIB | 0 | 0 | 60 |
| 41 | 70 | M | PD | G3 | II | 1 | 0 | 59 |
| 42 | 56 | F | PD | G3 | II | 0 | 0 | 74 |
| 43 | 78 | F | PD | G3 | IIIB | 0 | 0 | 39 |
| 44 | 65 | M | PD | G3 | III | 0 | 1 | 70 |
| 45 | 68 | M | PD | G3 | III | 1 | 1 | 69 |
| 46 | 57 | F | PD | G3 | IIIA | 0 | 0 | 61 |
| 47 | 68 | F | PD | G3 | III | - | - | - |
| 48 | 75 | F | PD | G3 | IV | - | - | 40 |
| 49 | 61 | M | PD | G3 | III | 1 | 0 | 70 |
| 50 | 55 | M | PD | G3 | III | - | - | - |
| 51 | 67 | F | PD | G3 | II | - | - | - |
| 52 | 50 | F | PD | G3 | III | - | - | - |
| 53 | 76 | F | UD | G4 | II | 0 | 0 | - |
| 54 | 51 | M | UD | G4 | II | - | - | 70 |

For grading, G1, G2, G3, and G4 represents well-differentiated (Low grade), moderately differentiated (Intermediate grade), poorly differentiated (High grade) and undifferentiated (High grade), respectively.
