# Supplementary material for: Gene-Expression Signatures Can Distinguish Gastric Cancer Grades and Stages
Source: PLoS One. 2011 Mar 18;6(3):e17819. doi: 10.1371/journal.pone.0017819 (PMC3060867; doi:10.1371/journal.pone.0017819)
Supplement: Table S6 — Recent key findings of biomarkers by transcriptomic and proteomic studies on gastric cancer. (DOCX) [file pone.0017819.s006.docx]

**Supplementary Table** 6: Recent key findings of biomarkers by transcriptomic and proteomic studies on gastric cancer

| **Reference** | **Genes (findings)** | **Techniques** | **Sample details** | **Category** |
| --- | --- | --- | --- | --- |
| ([Lee, Nam et al. 2010](#_ENREF_5)) | CDH17 | microarray | 450 tissues+502 independent | prognostic gene marker |
| ([Deng, Huang et al. 2010](#_ENREF_3)) | ER-alpha36 | RT-PCR, Western blot, immunohistochemistry | 22 cancer tissues | protein marker |
| ([Chen, Li et al. 2008](#_ENREF_2)) | TSPAN1, Ki67, CD34 | immunohistochemistry | 86 cancer tissues | cancer associated genes |
| ([Ishigami, Ueno et al. 2010](#_ENREF_4)) | CD133 | immunohistochemistry | 97 cancer tissues | prognostic protein marker |
| ([Bao, Qiao et al. 2010](#_ENREF_1)) | HMGB1 | immunohistochemistry | 76 cancer tissues | prognostic protein marker |
| ([Long, Ye et al. 2008](#_ENREF_6)) | nuclear factor kappa | immunohistochemistry | 60 cancer tissues | gene marker for stage IV |
| ([Yamada, Arao et al. 2008](#_ENREF_10)) | PDCD6 | microarray analysis | 40 tissues +19 independent | prognostic gene marker |
| ([Silva, Begnami et al. 2008](#_ENREF_7)) | E-cadherin, beta-catenin, and mucins (MUC1, MUC2, MUC5AC and MUC6) | microarray + immunohistochemistry | 62 young+ 453 old patients | gene markers |
| ([Takeno, Takemasa et al. 2008](#_ENREF_9)) | NEK6 and INHBA | microarray | 222 cancer tissues | gene/protein markers |
| ([Taddei, Castiglione et al. 2008](#_ENREF_8)) | NF2 | RT-PCR | 5 gastrointestinal stromal tumors | gene marker |

Bao, G., Q. Qiao, et al. (2010). "Prognostic value of HMGB1 overexpression in resectable gastric adenocarcinomas." World J Surg Oncol **8**(1): 52.

Chen, L., X. Li, et al. (2008). "Clinicopathological significance of overexpression of TSPAN1, Ki67 and CD34 in gastric carcinoma." Tumori **94**(4): 531-538.

Deng, H., X. Huang, et al. (2010). "A variant of estrogen receptor-alpha, ER-alpha36 is expressed in human gastric cancer and is highly correlated with lymph node metastasis." Oncol Rep **24**(1): 171-176.

Ishigami, S., S. Ueno, et al. (2010). "Prognostic impact of CD133 expression in gastric carcinoma." Anticancer Res **30**(6): 2453-2457.

Lee, H. J., K. T. Nam, et al. (2010). "Gene Expression Profiling of Metaplastic Lineages Identifies CDH17 as a Prognostic Marker in Early Stage Gastric Cancer." Gastroenterology.

Long, Y. M., S. Ye, et al. (2008). "Nuclear factor kappa B: a marker of chemotherapy for human stage IV gastric carcinoma." World J Gastroenterol **14**(30): 4739-4744.

Silva, E. M., M. D. Begnami, et al. (2008). "Cadherin-catenin adhesion system and mucin expression: a comparison between young and older patients with gastric carcinoma." Gastric Cancer **11**(3): 149-159.

Taddei, A., F. Castiglione, et al. (2008). "NF2 expression levels of gastrointestinal stromal tumors: a quantitative real-time PCR study." Tumori **94**(4): 551-555.

Takeno, A., I. Takemasa, et al. (2008). "Integrative approach for differentially overexpressed genes in gastric cancer by combining large-scale gene expression profiling and network analysis." Br J Cancer **99**(8): 1307-1315.

Yamada, Y., T. Arao, et al. (2008). "Identification of prognostic biomarkers in gastric cancer using endoscopic biopsy samples." Cancer Sci **99**(11): 2193-2199.
